# Real-time prognostic biomarkers for predicting in-hospital mortality and cardiac complications in COVID-19 patients

**DOI:** 10.1371/journal.pgph.0002836

**Published:** 2024-03-06

**Authors:** Rawan Omar, Sooyun Caroline Tavolacci, Lathan Liou, Dillan F. Villavisanis, Yoav Y. Broza, Hossam Haick

**Affiliations:** 1 Department of Chemical Engineering and Russell Berrie Nanotechnology Institute, Technion-Israel Institute of Technology, Haifa, Israel; 2 Graduate School of Biomedical Sciences, Icahn School of Medicine at Mount Sinai, New York, New York, United States of America; 3 Department of Medicine, Icahn School of Medicine at Mount Sinai, New York, New York, United States of America; University of Cape Town, SOUTH AFRICA

## Abstract

Hospitalized patients with Coronavirus disease 2019 (COVID-19) are highly susceptible to in-hospital mortality and cardiac complications such as atrial arrhythmias (AA). However, the utilization of biomarkers such as potassium, B-type natriuretic peptide, albumin, and others for diagnosis or the prediction of in-hospital mortality and cardiac complications has not been well established. The study aims to investigate whether biomarkers can be utilized to predict mortality and cardiac complications among hospitalized COVID-19 patients. Data were collected from 6,927 hospitalized COVID-19 patients from March 1, 2020, to March 31, 2021 at one quaternary (Henry Ford Health) and five community hospital registries (Trinity Health Systems). A multivariable logistic regression prediction model was derived using a random sample of 70% for derivation and 30% for validation. Serum values, demographic variables, and comorbidities were used as input predictors. The primary outcome was in-hospital mortality, and the secondary outcome was onset of AA. The associations between predictor variables and outcomes are presented as odds ratio (OR) with 95% confidence intervals (CIs). Discrimination was assessed using area under ROC curve (AUC). Calibration was assessed using Brier score. The model predicted in-hospital mortality with an AUC of 90% [95% CI: 88%, 92%]. In addition, potassium showed promise as an independent prognostic biomarker that predicted both in-hospital mortality, with an AUC of 71.51% [95% Cl: 69.51%, 73.50%], and AA with AUC of 63.6% [95% Cl: 58.86%, 68.34%]. Within the test cohort, an increase of 1 mEq/L potassium was associated with an in-hospital mortality risk of 1.40 [95% CI: 1.14, 1.73] and a risk of new onset of AA of 1.55 [95% CI: 1.25, 1.93]. This cross-sectional study suggests that biomarkers can be used as prognostic variables for in-hospital mortality and onset of AA among hospitalized COVID-19 patients.

## 1. Introduction

Coronavirus disease 2019 (COVID-19) pandemic is a significant global health crisis, with the number of cumulative cases exceeding 500 million and the death toll surpassing 6 million [1, 2]. Although acute symptoms of COVID-19 such as anosmia and respiratory complications are well-established, the assessment of potential systemic or long-term complications requires further investigation. Davis et al. showed that fatigue was the most common remaining symptom after 7 months and 30% of prevalence of tachycardia among the 966 COVID-19 confirmed cases, known as Long COVID [3]. Early studies demonstrated that the presence of atrial fibrillation and non-sustained ventricular arrhythmia was associated with 4.68 times and 8.92 times higher risk of Intensive Care Unit (ICU) admission, respectively [4].

Several studies have suggested possible underlying cardiac mechanisms during COVID-19 that cause cardiac complications. Cardiac injury was commonly found in COVID-19 hospitalized patients and correlated with elevated risk for in-hospital mortality [5–7]. Case report studies have shown that acute cardiac injury can lead to cardiac dysfunction, causing cardiogenic shock and increasing the probability of malignant arrhythmias [8]. Additional studies have reported that COVID-19 is associated with arrhythmia and myocarditis, heart failure, myocardial injury, and vascular inflammation [9–11]. Previous research has underscored the importance of measuring and evaluating cardiac biomarkers in hospitalized COVID-19 patients [9]. However, little attention has been paid to cardiac complications among hospitalized COVID-19 patients, and fewer studies have described employing biomarkers for examining these patients.

Several studies revealed a connection between high potassium levels and myocyte ischemia [12, 13], which triggers an imbalance of potassium levels, numerous inflammatory markers in the arrhythmogenesis pathway, and damages the myocardium that results in myocarditis and arrhythmias in COVID-19 [14]. Additional studies have shown that COVID-19 patients with high troponin T levels are at elevated risk for the development of severe disease, mortality, and require ICU admission [15, 16]. One study demonstrated that emerging arrhythmia and elevated creatine kinase (CK), creatine kinase-myocardial band (CK-MB), lactate dehydrogenase (LDH), and Interleukin-6 (IL-6) levels are associated with severe disease and ICU admission. Moreover, elevated levels of LDH hold prognostic value for mortality [16]. As a result, the study recommended that cardiac injury-related biomarkers be closely monitored in patients with COVID-19, especially in the acute phase of the disease.

Thus, it is of utmost importance to prioritize the surveillance of cardiac complications in COVID-19 patients during hospitalization, to facilitate earlier diagnosis of potential diseases, lower in-hospital mortality rates, and decrease the risk of cardiac complications. Quick, minimally invasive, real-time, and precise methods are warranted to monitor patients’ health continuously to provide accurate and early diagnosis of their condition.

This study selected specific biomarkers that could potentially aid in the creation of advanced technologies, like wearable sensor-based tools, to develop clinical support models for predicting mortality and cardiac complications among COVID-19 hospitalized patients [17–27]. The chosen biomarkers include serum potassium, serum magnesium, lactate, LDH, serum albumin, and troponin, which were hypothesized to have a significant impact on mortality and the onset of arrhythmias.

## 2. Methods

### 2.1 Study population

Data were collected from a total of 6,927 hospitalized patients with COVID-19 from March 1, 2020, to March 31, 2021, at one quaternary (Henry Ford Health) and five community hospital registries (Trinity Health Systems) [18]. Informed consent was waived because deidentified medical records were used. Assuming 24 candidate predictor parameters, an in-hospital mortality rate of 0.145, and a conservative 15% of the maximal Cox-Snell R^2^, we estimated that the minimum sample size for fitting the regression models was 4,199 with 609 events [28]. 4,881 patients (70%), the training set, were used to build a predictive model and the outcome was analyzed in a holdout validation set of 2,046 patients (30%). All diagnoses including atrial arrhythmias (AA) (atrial fibrillation (AF) and atrial flutter), co-morbid conditions, and in-hospital mortality were defined with 10^th^ revision of International Classification of Diseases (ICD-10) codes from deidentified electronic health records [17, 18]. This study adhered to the Transparent Reporting of a Multivariable Prediction Model for Individual Prognosis or Diagnosis (TRIPOD) reporting guideline [29].

### 2.2 Prognostic biomarkers

The primary outcome of interest was in-hospital mortality, and the secondary outcome of interest was new-onset AA. New onset was defined as having no previous ICD-10 code diagnosis [18]. Age, gender, race, BMI, diabetes mellitus (DM), congestive heart failure (CHF), pulmonary embolism (PE), solid cancer, hematological malignancy (HEMA), and 16 biomarkers were chosen based on their established clinical relevance and physiological significance, due to their suitability and applicability in development of online monitoring tools and wearable devices [5, 14, 16, 30]. LDH (U/L), ferritin(ng/ml), troponin I (ng/ml), creatine phosphokinase (CPK) (U/L), c-reactive protein (CRP) (mg/dl), B-type natriuretic peptide (BNP) (pg/ml), serum creatine (Cr) (mg/dl), lactate (mmol/L), serum potassium (pK and lK) (mEq/L), serum magnesium (pMg and lMg) (mg/dl), serum albumin (Albu) (g/dl), hemoglobin (Hb) (gm/dl), diastolic and systolic blood pressure (DBP and SBP) (mmHg) (p = peak, l = lowest)) were included as predictor variables to build the model. In the original data set, all parameters except for Albu and Hb were recorded at their highest value. Potassium was investigated in greater depth due to its statistical significance during exploratory data analysis and potential clinical relevance. The missing data were handled by multiple imputations by chained equations under the missing at random assumption and performed using the “mice” package in R [31, 32].

### 2.3 Statistical analysis

Age, BMI, demographics, hospital events, and 16 biomarkers were treated as continuous variables and summarized using mean, standard deviation (SD), median, and interquartile range (IQR) in **Table 1**. Meanwhile, gender, race, and comorbidities were considered categorical variables and expressed as frequency and percentage. The Mann-Whitney U test was employed to identify median differences in peak serum potassium for each outcome. Initially, a univariate logistic regression was conducted to analyze the primary outcome (in-hospital mortality) and secondary outcome (new onset of AA). A randomly selected 70% sample was used to derive the multivariable logistic regression model, with the remaining 30% used for validation. All predictors described above were included in the final model. The associations between the predictor variables and outcome are presented as odds ratio (OR) with 95% confidence intervals (CIs) in **Table 2**. Multicollinearity was assessed using a correlation plot (**S1 Fig**). Nagelkerke R^2^ was used as a measure of pseudo-R^2^ to assess the goodness of fit. Discrimination was assessed using area under ROC curve (AUC). Calibration was assessed using the Brier score. All performance statistics reported were calculated using the holdout validation set. We compared the models for the clinical utility using decision curve analysis [33]. This analysis assesses the trade-off between the potential harms and the benefits of true positives and that may arise from false positives across a range of threshold probabilities. Each model was compared using the two default scenarios of treat all or treat none, with the mean model prediction used for each individual. This approach implicitly considers both discrimination and calibration and extends model evaluation to consider the ramifications on clinical decision-making [34]. A 2-sided *p*-value less than 0.05 was considered statistically significant. All statistical analysis were conducted using SAS 9.4 software (SAS Institute, USA) and GraphPad Prism 9 (GraphPad Software, USA).

**Table 1:** Baseline demographic, biomarker, and hospital event characteristics.

	Overall
	(N = 6927)
**Demographics**	
**Age**	
Mean (SD)	65.2 (16.7)
Median [Min, Max]	67.0 [21.0, 90.0]
**BMI (kg/m2)**	
Mean (SD)	31.2 (8.47)
Median [Min, Max]	29.7 [2.34, 80.7]
**Gender**	
Female	3514 (50.7%)
Male	3413 (49.3%)
**Race**	
Black	2404 (34.7%)
Other	448 (6.5%)
White	3839 (55.4%)
Missing	236 (3.4%)
**Comorbidities**	
**Diabetes Mellitus**	
No	4447 (64.2%)
Yes	2480 (35.8%)
**Hypertension**	
No	2323 (33.5%)
Yes	4604 (66.5%)
**Congestive Heart Failure**	
No	5671 (81.9%)
Yes	1256 (18.1%)
**History of Coronary Artery Disease**	
No	3798 (54.8%)
Yes	531 (7.7%)
**History of Stroke/Transient Ischemic Attack**	
No	6236 (90.0%)
Yes	691 (10.0%)
**History of Deep Vein Thrombosis**	
No	6550 (94.6%)
Yes	377 (5.4%)
**History of Pulmonary Embolism**	
No	6669 (96.3%)
Yes	258 (3.7%)
**History of Pulmonary Disease** ^a^	
No	5345 (77.2%)
Yes	1582 (22.8%)
**History of Liver Disease** ^b^	
No	6754 (97.5%)
Yes	173 (2.5%)
**History of Chronic Kidney Disease**	
No	6025 (87.0%)
Yes	902 (13.0%)
**History of End-Stage Renal Disease**	
No	6717 (97.0%)
Yes	210 (3.0%)
**History of Malignancies** ^c^	
No	5837 (84.3%)
Yes	1090 (15.7%)
**Biomarkers**	
**Peak Lactate dehydrogenase (U/L)**	
Mean (SD)	381 (402)
Median [Min, Max]	318 [69.0, 9750]
**Peak Ferritin (ng/mL)**	
Mean (SD)	848 (2140)
Median [Min, Max]	495 [5.00, 78700]
**Peak Troponin-I (ng/mL)**	
Mean (SD)	0.198 (1.02)
Median [Min, Max]	0.0280 [0.00400, 18.9]
**Peak Creatine phosphokinase (U/L)**	
Mean (SD)	452 (10200)
Median [Min, Max]	87.0 [10.0, 694000]
**Peak C-reactive protein (mg/dL)**	
Mean (SD)	7.88 (6.42)
Median [Min, Max]	7.30 [0.100, 48.6]
**Peak B-type natriuretic peptide (pg/ml)**	
Mean (SD)	221 (433)
Median [Min, Max]	72.0 [5.00, 3990]
**Peak Serum Creatinine (mg/dL)**	
Mean (SD)	1.84 (2.02)
Median [Min, Max]	1.14 [0.230, 26.3]
**Peak Serum Lactate (mmol/L)**	
Mean (SD)	2.19 (1.97)
Median [Min, Max]	1.60 [0.300, 29.1]
**Peak Serum Potassium (mEq/L)**	
Mean (SD)	4.67 (0.780)
Median [Min, Max]	4.50 [2.50, 9.70]
**Lowest Serum Potassium (mEq/L)**	
Mean (SD)	3.60 (0.542)
Median [Min, Max]	3.60 [1.20, 22.0]
**Peak Serum Magnesium (mg/dL)**	
Mean (SD)	2.29 (0.419)
Median [Min, Max]	2.20 [1.00, 9.50]
**Lowest Serum Magnesium (mg/dL)**	
Mean (SD)	1.84 (0.284)
Median [Min, Max]	1.80 [0.500, 5.90]
**Lowest Serum Albumin (g/dL)**	
Mean (SD)	2.92 (0.613)
Median [Min, Max]	2.90 [1.00, 5.70]
**Lowest Hemoglobin (g/dL)**	
Mean (SD)	11.5 (1.88)
Median [Min, Max]	11.5 [1.90, 19.4]
**Presenting Systolic Blood Pressure (mmHg)**	
Mean (SD)	134 (25.4)
Median [Min, Max]	132 [0, 266]
**Presenting Diastolic Blood Pressure (mmHg)**	
Mean (SD)	74.9 (15.9)
Median [Min, Max]	74.0 [0, 235]
**Hospital Events**	
**In-Patient Mortality**	
No	5842 (84.3%)
Yes	1085 (15.7%)
**ICU Admission**	
No	5350 (77.2%)
Yes	1577 (22.8%)
**Hospital Readmission**	
No	6312 (91.1%)
Yes	615 (8.9%)
**Hospital Readmission within 90 days**	
No	6341 (91.5%)
Yes	586 (8.5%)
**Respiratory Failure Requiring Mechanical Ventilation**	
No	6081 (87.8%)
Yes	846 (12.2%)
**New Onset Heart Failure**	
No	6636 (95.8%)
Yes	291 (4.2%)
**Transient Ischemic Attack/Ischemic Stroke**	
No	6768 (97.7%)
Yes	159 (2.3%)
**Acute Renal Failure**	
No	4594 (66.3%)
Yes	2333 (33.7%)
**Ventricular Fibrillation**	
No	6905 (99.7%)
Yes	22 (0.3%)
**Ventricular Tachycardia**	
No	6750 (97.4%)
Yes	177 (2.6%)
**Type of Atrial Arrhythmia**	
History of Atrial Arrhythmias	779 (11.2%)
New-onset Atrial Arrhythmias	626 (9.0%)
Normal Sinus Rhythm	5522 (79.7%)

^a^History of COPD, asthma, bronchiectasis, and interstitial lung disease

^b^History of alcoholic liver disease, non-alcoholic steatohepatitis, hepatitis B, and hepatitis C

^c^History of cancer, leukemia, and hepatocellular carcinoma

**Table 2:** Univariate associations between variables and in-hospital mortality.

Variable	OR (95% CI)^b^
Age	1.07 (1.06, 1.07)
Female (Ref: Male)	0.65 (0.56, 0.76)
Black race (Ref: White)	0.71 (0.60, 0.84)
Other race (Ref: White)	0.46 (0.31, 0.66)
Diabetes Mellitus	1.10 (0.94, 1.33)
Congestive heart failure	2.30 (1.93, 2.73)
History of Pulmonary Embolism	1.20 (0.82, 1.76)
History of Malignancies^a^	1.89 (1.57, 2.28)
BMI	0.95 (0.94, 0.96)
Presenting Systolic blood pressure	0.99 (0.99, 1.00)
Peak Lactate dehydrogenase (U/L)	1.00 (1.00, 1.00)
Peak Ferritin (ng/mL)	1.00 (1.00, 1.00)
Peak Troponin-I (ng/mL)	1.18 (1.11, 1.25)
Peak Creatine phosphokinase (U/L)	1.00 (1.00, 1.00)
Peak C-reactive protein (mg/dL)	1.09 (1.08, 1.11)
Peak B-type natriuretic peptide (pg/ml)	1.00 (1.00, 1.00)
Peak Serum creatinine (mg/dL)	1.31 (1.26, 1.35)
Peak Lactate (mmol/L)	1.54 (1.46, 1.62)
Peak Serum potassium (mEq/L)	2.53 (2.30, 2.79)
Peak Serum magnesium (mg/dL)	5.51 (4.56, 6.67)
Lowest Albumin (g/dL)	0.12 (0.11, 0.15)
Lowest Hemoglobin (g/dL)	0.77 (0.74, 0.80)

^a^History of cancer, leukemia, and hepatocellular carcinoma

^b^Odds ratio (OR) with 95% confidence intervals (CIs)

### 2.4 Ethical statement

This study used publicly available data in Mendeley (DOI:**10.17632/rm6rjpft8j.5**) from a published study.[18] The original study was approved “The study was approved as a retrospective study by institutional review boards at Henry Ford Health System (protocol # 13785) and Trinity Health System (protocol # 2021–009). The need for informed consent was waived for the use of deidentified medical records”.

## 3. Results

### 3.1 Clinical characteristics

A total of 6,927 hospitalized patients with COVID-19 were evaluated. The mean age was 65.2 ± 16.7 years, 50.7% were women and 55.4% were white. 35.8% of patients had diabetes, 18.1% were with congestive heart failure, 3.7% had a history of pulmonary embolism, and 15.7% had a history of malignancies. Summary statistics (mean, SD, and proportion) of variables used in the model are summarized in **Table 1**.

### 3.2 Potassium as a potential biomarker

**Fig 1:**
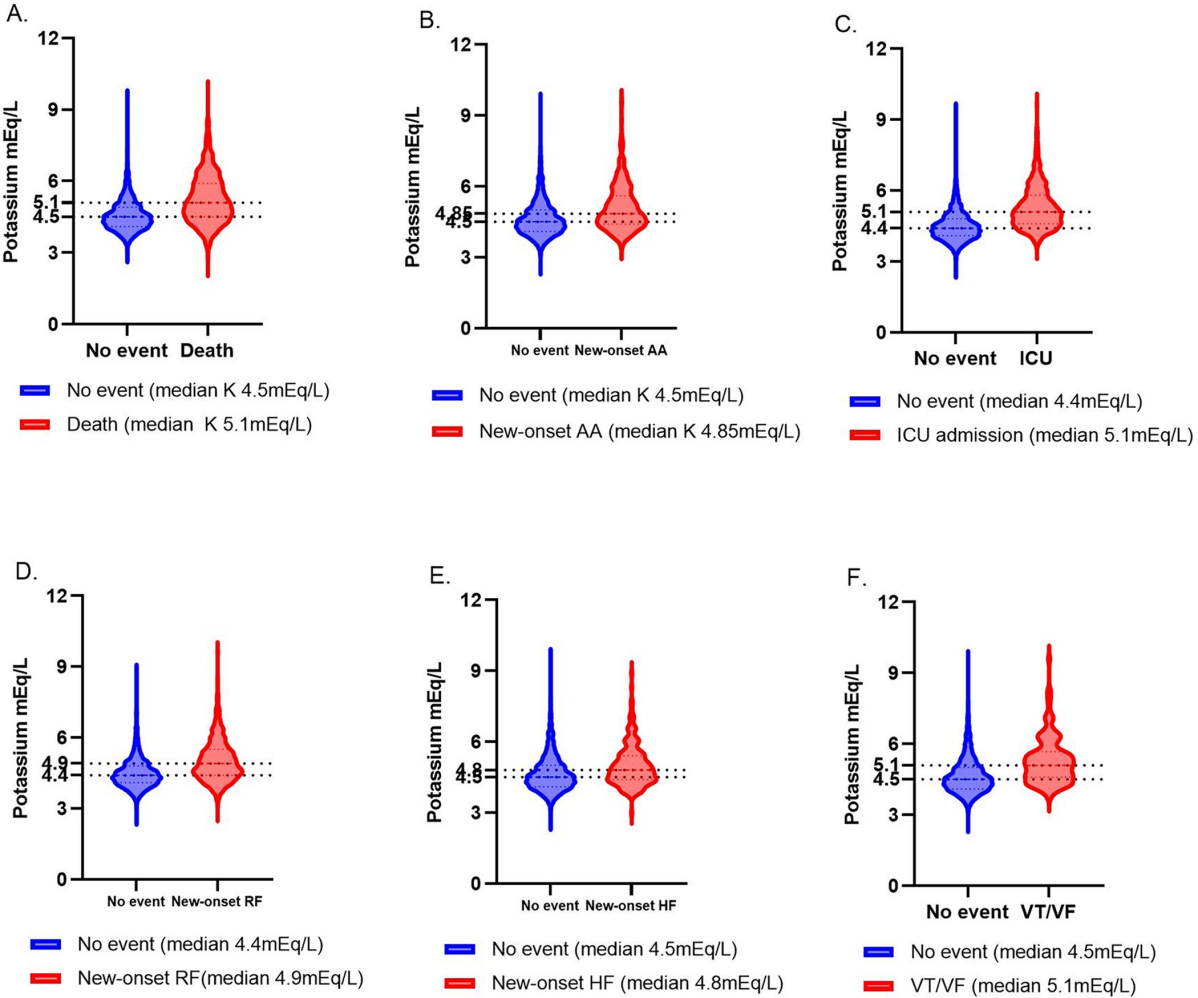
Differences in median potassium values across different outcomes. (A) No event (n = 4802) vs death (n = 897); median 4.5 mEq/L vs 5.1 mEq/L (p<0.0001, Mann-Whitney U test). (B) No event (Normal sinus rhythm and History of AA) (n = 5171) vs New-onset of AA (AA) (n = 528); median 4.5 mEq/L vs 4.85 mEq/L (p<0.0001, Mann-Whitney U test). (C) No event (n = 4387) vs ICU admission (n = 1312); median 4.4 mEq/L vs 5.1 mEq/L (p<0.0001, Mann-Whitney U test). (D) No event (n = 3726) vs New-onset of RF (n = 1973); median 4.4 mEq/L vs 4.9 mEq/L (p<0.0001, Mann-Whitney U test). (E) No event (n = 5477) vs New-onset of HF (n = 222); median 4.5 mEq/L vs 4.8 mEq/L (p<0.0001, Mann-Whitney U test). (F) No event (n = 5563) vs New-onset of VT/VF (n = 136); median 4.5 mEq/L vs 5.1 mEq/L (p<0.0001, Mann-Whitney U test).

To evaluate the prognostic capacity of potassium as a biomarker of in-hospital mortality and onset of AA, we initially conducted a screening analysis (n = 5,110) using our full sample with available information on potassium, comparing potassium levels between patients who died and those who survived. Serum peak potassium (pK) showed significant differences in relation to both primary (in-hospital mortality) and secondary (new onset of AA) outcomes, [event of death (n = 740, 14.5%); new onset of AA (n = 528, 10.3%)]. Potassium was also significantly different in other hospital severe events including event of pulseless ventricular tachycardia/ventricular fibrillation (VT/VF) (n = 103, 2%); new onset of heart failure (HF) (n = 196, 3.8%); new onset of renal failure (RF) (n = 1704, 33.3%); and ICU admission (n = 1159, 22.7%; 5.1 mEq/L] (p<0.0001, Mann-Whitney *U* test) (**Fig 1**). An unadjusted increase of 1 mEq/L of potassium was associated with an in-hospital mortality risk of 2.53 [95% CI: 2.30, 2.79] and a risk of new onset of AA of 1.70 [95% CI: 1.53, 1.89]. Serum potassium levels predicted in-hospital mortality with an AUC of 70.91% [95% Cl: 68.81%, 73.01%] and new onset of AA with an AUC of 64.54% [95% Cl: 61.89%, 67.19%]. (**S1 Table**).

### 3.3 Performance of prediction model for in-hospital mortality

**Fig 2:**
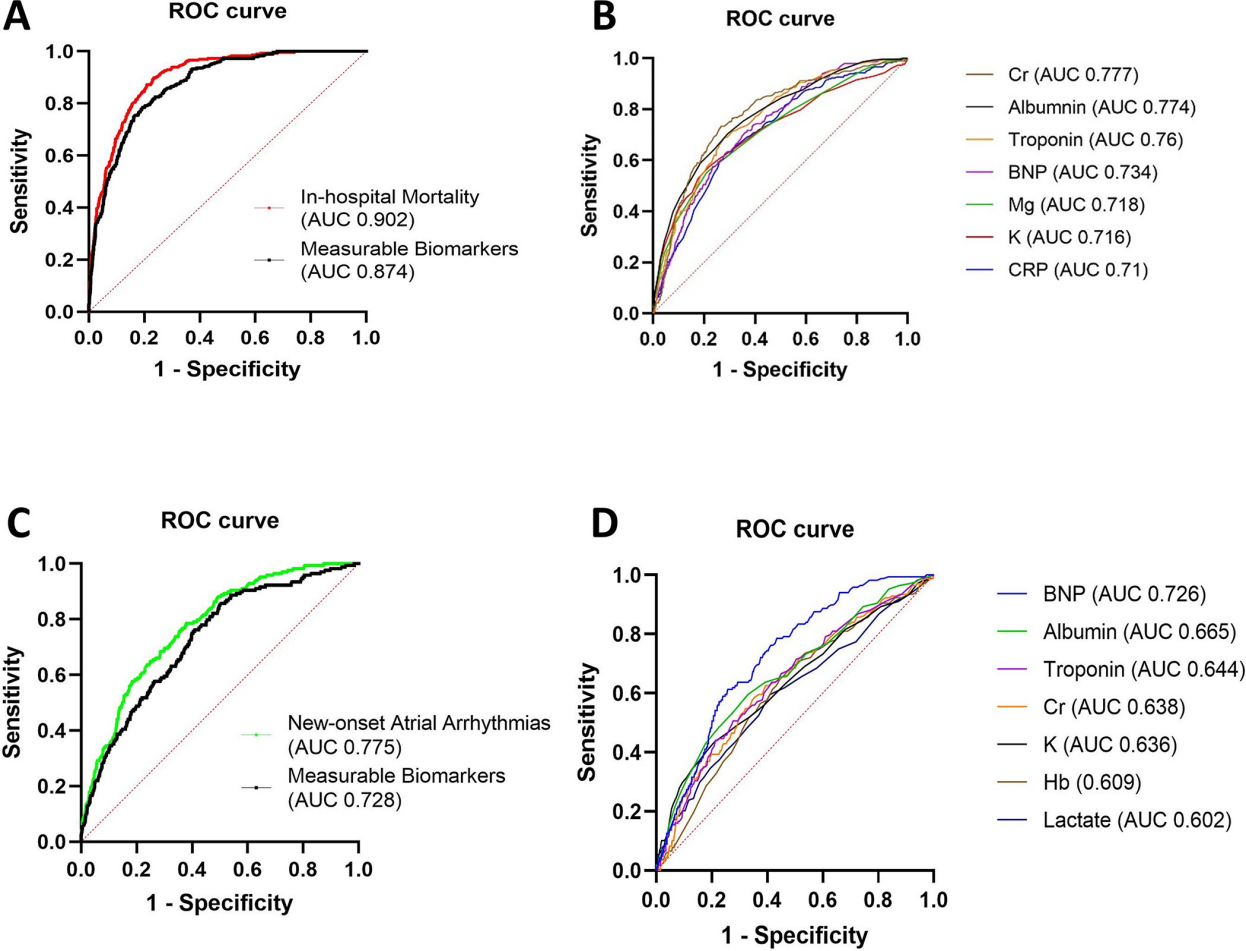
ROC for predictive models. (A) In-hospital mortality: all biomarkers and measurable biomarkers; demographics (age, gender, race), medical history (diabetes mellitus, congestive heart failure, pulmonary Embolism, Malignancies), measurable biomarkers (BMI, LDH, Ferritin, Troponin, CPK, CRP, BNP, Cr, Lactate, K, Mg, Hb, SBP were used to predict the outcome In-hospital mortality. (B) Individual biomarkers; measurable biomarkers AUC≥0.7 was reported (C) New-onset AA: All biomarkers and measurable biomarkers; demographics (age, gender, race), medical history (diabetes mellitus, congestive heart failure, pulmonary Embolism, Malignancies), measurable biomarkers (BMI, LDH, Ferritin, Troponin, CPK, CRP, BNP, Cr, Lactate, K, Mg, Hb, SBP were used to predict the outcome new-onset AA. (D) Individual Biomarkers; measurable biomarkers AUC≥0.6 was reported.

In order to build a model for predicting in-hospital mortality, three components were considered based on their clinical importance for predicting in-hospital mortality. Demographics (age, gender, and race), comorbidities (diabetes, congestive heart failure, history of pulmonary embolism, and malignancies), and measurable biomarkers (BMI, LDH, ferritin, troponin, CPK, CRP, BNP, Cr, lactate, pK, pMg, lAlbu, lHb, and SBP (*p = peak, l = lowest)) were included in the multivariable logistic regression (**Fig 2**).

The final mortality model (model_mortality_) predicted in-hospital mortality with a validation AUC of 0.90 [95% CI: 0.88, 0.92] (**Table 3**). The model_mortality_ had a specificity of 0.96, PPV of 0.63, and an NPV of 0.90 at a threshold of 0.5. The model_mortality_ had a Brier score of 0.08. Full model coefficients with 95% confidence intervals are summarized in **S2 Table**. Age [OR = 1.07, 95% CI: 1.05, 1.08], CRP [OR = 1.06, 95% CI: 1.03, 1.08], creatinine [OR 1.13, 95% CI: 1.03, 1.24], lactate [OR = 1.33, 95% CI: 1.20, 1.47], magnesium [OR = 3.35, 95% CI: 2.33, 4.82] were significant predictors of mortality. Albumin [OR = 0.37, 95% CI: 0.27, 0.52] was significantly associated with decreased odds of mortality. The biomarker-only model predicted mortality with an AUC of 0.87 [95% CI: 0.85, 0.89]. Biomarker-only model coefficients are summarized in **S3 Table**. Improvement of model discrimination is shown in **Fig 2A**, comparing the model_mortality_ vs. the biomarkers-only model. The ROC of each independently significant biomarker is shown in **Fig 2B and S4 Table**. Decision curve analysis showed that net benefit using the predictive model was better than treating all or none across a range of reasonable threshold probabilities (**S2 Fig**).

**Table 3:** Performance for biomarkers-only model and full model for in-hospital mortality.

Model	AUC (95% CI)	Brier Score	Nagelkerke’s R^2^	Sensitivity	Specificity
Biomarkers Only	0.874 (0.8555, 0.893)	0.090	0.38	0.33	0.97
Full	0.902 (0.885, 0.919)	0.08	0.46	0.37	0.96

### 3.4 Performance of prediction models for secondary outcomes

The final model for new onset of AA (model_AA_) had an AUC of 0.77 [95% CI: 0.74, 0.81] (**Fig 2C and S5 Table**). The model_AA_ had a specificity of 0.99, PPV of 0.60, and NPV of 0.92 at a cutoff threshold of 0.5. Individual biomarkers were also used to predict onset AA as presented in **Fig 2D**. The ROC curve for the model_AA_ adjusting for type of AA which included history of AA and new onset of AA was also presented in **S3 Fig**. Age [OR = 1.04, 95% CI: 1.02, 1.06], lactate [OR = 1.13, 95% CI: 1.02, 1.25], potassium [OR = 1.55, 95% CI: 1.25, 1.93] were significant predictors of mortality. Albumin [OR = 0.66, 0.47, 0.93] was significantly associated with decreased odds of onset AA. Model_AA_ and biomarker-only model coefficients with 95% confidence intervals are summarized in **S6** and **S7 Tables**. Additionally, increased serum Mg level was strongly associated with both ICU admission (OR = 4.48 [95% CI: 3.52, 5.71]) and new onset of RF (OR = 2.40 [95% CI: 1.78, 3.25]), which was not significantly associated with mortality nor new onset of AA (**S8** and **S9 Tables**). Additional models developed using the same set of predictor variables could predict ICU admission (AUC of 0.86 [95% CI: 0.84, 0.88]) and the new onset of RF (AUC of 0.78 [95% CI: 0.75, 0.80]) (**S4** and **S5 Figs**).

## 4. Discussion

In this cross-sectional study of 6,927 hospitalized patients due to COVID-19, we developed a model including biomarkers and baseline demographic variables to predict the in-hospital mortality and incidence of cardiac complications. Among the tested measurable biomarkers, potassium also predicted the outcomes independently, showing a robust association with in-hospital death rate and the presence of AA.

COVID-19 has been the cause of numerous hospitalizations and fatalities across the world [1]. Although it is widely thought that cardiac arrhythmias are sequelae of COVID-19, new studies suggest there may be underlying causes in the heart that lead to such issues [10, 15]. Cardiac injury has been observed frequently in hospitalized COVID-19 patients and is associated with higher risk of in-hospital mortality [5]. Despite the significance of cardiac complications in hospitalized COVID-19 patients, there has been limited research on identifying potential biomarkers to predict these outcomes. Additionally, few studies have investigated the use of health indicators to examine these patients, despite the potential for these indicators to predict both severe clinical courses and cardiac complications in hospitalized COVID-19 patients [9]. Herein, our full model mainly included co-morbidities and measurable biomarkers (LDH, ferritin, troponin I, CPK, CRP, BNP, Cr, lactate, potassium, Mg, Albu, Hb, and SBP). Measurable biomarkers were utilized to develop a biomarkers-only model due to their established clinical and physiological relevance. These biomarkers, known for their predictability and accuracy, can often outperform binary medical parameters and can be incorporated into monitoring tools, such as wearables [5, 14, 16, 30, 35]. Based on our findings, our model exhibited excellent discrimination in predicting in-hospital mortality, with an AUC of 0.902 and a specificity of 0.96. It relies on a predictive model similar to the modern concept of deep learning-based models that use electronic health records [36], outperforming conventional clinical tools such as the ’traditional risk scores’. These conventional approaches incorporate variables that are usually assessed in clinical settings, such as lipid profile, blood pressure, glucose levels and history of smoking [37–39]. Some of these risk scores include the augmented Early Warning Score (aEWS) [36], QRISK3 [40], American College of Cardiology/American Heart Association (ACC/AHA) risk scores [41], Framingham risk score (FRS) [42], SCORE [43], and the United Kingdom Prospective Diabetes Study 60 (UKPDS60) [44]. In addition, several previous models for predicting COVID-19 in-hospital mortality have been developed. For example, a risk score system has been developed based on complete blood count and age [45]. Another model was developed using data from 452 COVID-19 patients at the age of 60 and included lymphopenia, D-dimer, coronary heart disease and procalcitonin [46]. Previous studies have emphasized the significance of monitoring mortality rates among COVID-19 patients to prioritize hospitalization and provide timely medical care, ultimately reducing the number of deaths [47, 48].

Using the same set of predictor variables, we also developed a model that predicted the new onset of AA in hospitalized COVID-19 patients. This model predicted the onset of AA with AUC of 0.77 and a specificity of 0.99. Previous literature pointed out the significance of predicting cardiac issues in COVID-19 patients. Early studies in the first China patient cohorts reported 17% of patients suffering from cardiac arrhythmia, with rates up to 44% in ICU patients [49]. Another study found similar rates of arrhythmia events in COVID-19 patients hospitalized in ICU, with the most common arrhythmic event of AF [4]. One study found that cardiac arrhythmia was the most common cardiac event associated with COVID-19 hospitalization, and concluded that the high incidence of arrhythmias, as well as their potential prognostic implications, make it necessary to screen patients with risk factors [50].

Among the chosen biomarkers, we picked potassium for screening analysis, considering the inherent importance of electrolytes in diagnostics and the clinical role of potassium as a health indicator [12, 13, 51–56]. Furthermore, several studies have been performed using next-generation platforms, showing the differential expression of biomarkers, including omic biomarkers or protein and gene expression of these biomarkers and others related to potassium, in the context of mortality or cardiac complications [57–65]. While biomarkers such as troponin and albumin have established correlations with cardiac function, predicting mortality and AA, we opted to focus on potassium as a singular biomarker to test its predictability for different outcomes. Based on our findings, potassium showed promise as an independent biomarker that predicted both hospital mortality and the onset of AA. These findings support the fact that potassium is a significant prognostic biomarker for in-hospital mortality and the onset of AA following the previous literature that described the relationship between elevated levels of potassium and health deterioration and increased mortality [55]. In previous studies, potassium showed a strong correlation with arrhythmia and mortality especially since abnormalities in electrolytes are considered common among COVID-19 patients [51, 66–68]. In addition, abnormal levels of potassium were associated with in-hospital mortality and arrhythmia among patients admitted with suspected ACS [56]. Generally, electrolyte biomarkers have garnered interest for the development of wearable devices designed to estimate risk of cardiac complications and in-hospital death. Accordingly, it’s important to monitor these biomarkers and employ them in health diagnosis, to predict diseases severity and mortality [51–54].

By monitoring prognostic biomarkers in real-time for in-hospital mortality and the onset of AA in hospitalized COVID-19 patients, it is possible to gain rapid and precise estimations of health status which can be used to avert complications and mortality. In addition, utilizing wearable devices sent home with discharged patients would offer a cost-efficient method for independent, long-term health surveillance [69–73]. Our constructed prediction model using prognostic biomarkers was able to predict in-hospital mortality and cardiac problems for COVID-19 patients. Following on our study, these prognostic biomarkers may prove valuable for building a prediction model not only in the case of COVID-19 patients but also in other patient cohorts; thus further investigations are warranted.

The current study has several limitations; First, our models were validated only with an internal validation set, which limits external generalization. Second, model development depended on peak-measured biomarkers since we did not have access to serial measurements, but the inclusion of serial measurements may be more powerful [74]. Third, given the retrospective nature of our dataset, no coefficient within the model has a causal interpretation. Fourth, we used a multiple imputation approach to impute missing data, which relies on missing data at random assumptions. Fifth, the model relies on predictors that are measured invasively, in contrast to other non-invasive methods such as breath analysis, which comes with constrains and limits the usage of the model in routine settings. Sixth, there’s a lack of comparison between the developed model and existing models designed for the same usage. For robustness, a bootstrapping approach could be considered to minimize bias; however, this was not considered for the current study due to computational overhead. In general, multiple imputation is more statistically powerful for model development than throwing out data in a complete case approach [31].

## 5. Conclusions

Our current research outlines a prognostic biomarker-based predictive model for in-hospital mortality and atrial arrhythmia. Among the measurable biomarkers tested, potassium proved to be a valuable independent indicator in forecasting both mortality and AA. Going forward, further investigations should continue to assess the predictive capacity of biomarkers in other patient populations. Moreover, future research endeavors may involve utilizing these prediction models to construct wearable, real-time monitoring devices that can assist in more informed clinical decision-making and online health tracking for patients.

## Supporting information

S1 Checklist STROBE statement—Checklist of items that should be included in reports of observational studies (cross-sectional study). (DOCX)

S1 Table Performance of potassium for in-hospital mortality and atrial arrhythmia. (PDF)

S2 Table Coefficients of full model for in-hospital mortality. (PDF)

S3 Table Coefficients of biomarker-only model for in-hospital mortality. (PDF)

S4 Table AUC model discrimination for individual biomarkers. (PDF)

S5 Table Performance for biomarkers-only model and full model for new-onset atrial arrhythmia. (PDF)

S6 Table Coefficients of full model for new-onset atrial arrhythmia. (PDF)

S7 Table Coefficients of biomarker-only model for new-onset atrial arrhythmia. (PDF)

S8 Table Odds ratios of biomarker-only model for ICU admission. (PDF)

S9 Table Odds ratios of biomarker-only model for renal failure. (PDF)

S1 Fig Parameter covariance heatmap. (PDF)

S2 Fig Decision curve analysis for full model for in-hospital mortality. (PDF)

S3 Fig ROC for model adjusting for type of atrial arrhythmia. (PDF)

S4 Fig ROC for ICU admission. (PDF)

S5 Fig ROC for new-onset renal failure. (PDF)
