# Supplementary material for: Real-time prognostic biomarkers for predicting in-hospital mortality and cardiac complications in COVID-19 patients
Source: PLOS Glob Public Health. 2024 Mar 6;4(3):e0002836. doi: 10.1371/journal.pgph.0002836 (PMC10917247; doi:10.1371/journal.pgph.0002836)
Supplement: S1 Table — (PDF) [file pgph.0002836.s002.pdf]

Table S1. Performance of Potassium for In-Hospital Mortality and Atrial Arrhythmia

Outcome	OR (95% CI)	AUC (95% CI)
In-hospital mortality	2.53 (95% CI: 2.30 – 2.79)	70.91% (95% CI: 68.81%, 73.01%)
AA	1.70 (95% CI: 1.53 – 1.89)	64.54% (95% Cl: 61.89 67.19)
