# Supplementary material for: Real-time prognostic biomarkers for predicting in-hospital mortality and cardiac complications in COVID-19 patients
Source: PLOS Glob Public Health. 2024 Mar 6;4(3):e0002836. doi: 10.1371/journal.pgph.0002836 (PMC10917247; doi:10.1371/journal.pgph.0002836)
Supplement: S2 Table — (PDF) [file pgph.0002836.s003.pdf]

Table S2. Coefficients of Full Model for In-Hospital Mortality

Variable	Beta coefficient	95% CI	
Age	0.0705	0.0607	0.0803
Female (Ref: Male)	-0.1016	-0.2047	0.0014
Black race (Ref: White)	-0.0705	-0.2795	0.1386
Other race (Ref: White)	-0.2238	-0.5497	0.1022
Diabetes Mellitus	-0.1217	-0.2284	-0.0151
Congestive heart failure	0.0138	-0.1079	0.1355
History of Pulmonary Embolism	0.0240	-0.2185	0.2666
History of Malignancies[a]	0.0462	-0.0749	0.1673
BMI	-0.0108	-0.0258	0.0043
Peak Lactate dehydrogenase (U/L)	0.0007	0.0003	0.0011
Peak Ferritin (ng/mL)	0.0000	0.0000	0.0001
Peak Troponin-I (ng/mL)	0.1263	0.0521	0.2006
Peak Creatine phosphokinase (U/L)	-0.0001	-0.0001	0.0000
Peak C-reactive protein (mg/dL)	0.0616	0.0482	0.0750
Peak B-type natriuretic peptide (pg/ml)	0.0004	0.0002	0.0006
Peak Serum Creatinine (mg/dL)	0.1484	0.1010	0.1958
Peak Lactate (mmol/L)	0.2903	0.2266	0.3541
Peak Serum potassium (mEq/L)	0.3230	0.1845	0.4614
Peak Serum magnesium (mg/dL)	0.8189	0.5861	1.0517
Lowest Albumin (g/dL)	-1.3118	-1.5221	-1.1015
Lowest Hemoglobin (g/dL)	0.0599	-0.0021	0.1218
Presenting Systolic blood pressure (mmHg)	-0.0028	-0.0065	0.0009

[a]History of COPD, asthma, bronchiectasis, and interstitial lung disease
