# Supplementary material for: Real-time prognostic biomarkers for predicting in-hospital mortality and cardiac complications in COVID-19 patients
Source: PLOS Glob Public Health. 2024 Mar 6;4(3):e0002836. doi: 10.1371/journal.pgph.0002836 (PMC10917247; doi:10.1371/journal.pgph.0002836)
Supplement: S3 Table — (PDF) [file pgph.0002836.s004.pdf]

Table S3. Coefficients of Biomarker-Only Model for In-Hospital Mortality

Variable	Beta coefficient	95% CI	
BMI	-0.0543	-0.0676	-0.0410
Peak Lactate dehydrogenase (U/L)	0.000317	-0.00001	0.000648
Peak Ferritin (ng/mL)	0.000021	-0.00002	0.000064
Peak Troponin-I (ng/mL)	0.0766	0.00948	0.1438
Peak Creatine phosphokinase (U/L)	-0.00006	-0.00012	-4.78E-6
Peak C-reactive protein (mg/dL)	0.0607	0.0479	0.0736
Peak B-type natriuretic peptide (pg/ml)	0.000546	0.000364	0.000727
Peak Serum Creatinine (mg/dL)	0.1078	0.0656	0.1499
Peak Lactate (mmol/L)	0.3009	0.2400	0.3617
Peak Serum potassium (mEq/L)	0.2934	0.1641	0.4227
Peak Serum magnesium (mg/dL)	0.7444	0.5321	0.9568
Lowest Albumin (g/dL)	-1.4414	-1.6359	-1.2469
Lowest Hemoglobin (g/dL)	0.0599	0.00279	0.1169
Presenting Systolic blood pressure (mmHg)	-0.00207	-0.00562	0.00148
