# Supplementary material for: Real-time prognostic biomarkers for predicting in-hospital mortality and cardiac complications in COVID-19 patients
Source: PLOS Glob Public Health. 2024 Mar 6;4(3):e0002836. doi: 10.1371/journal.pgph.0002836 (PMC10917247; doi:10.1371/journal.pgph.0002836)
Supplement: S4 Table — (PDF) [file pgph.0002836.s005.pdf]

Table S4. AUC Model Discrimination for Individual Biomarkers

In-hospital Mortality	New-Onset Atrial Arrhythmias
Cr (AUC 0.777) (95% Cl 0.749 – 0.806)	BNP (AUC 0.726) (95% Cl 0.691 – 0.762)
Albumin (AUC 0.774) (95% Cl 0.746 – 0.803)	Albumin (AUC 0.665) (95% Cl 0.621 – 0.71)
Troponin (AUC 0.76) (95% Cl 0.732 – 0.787)	Troponin (AUC 0.644) (95% Cl 0.599 – 0.688)
BNP (AUC 0.734) (95% Cl 0.705 – 0.763)	Cr (AUC 0.638) (95% Cl 0.594 – 0.682)
Mg (AUC 0.718) (95% Cl 0.686 – 0.751)	K (AUC 0.636) (95% Cl 0.589 – 0.683)
K (AUC 0.716) (95% Cl 0.681 – 0.751)	Hb (0.609) (95% Cl 0.565 – 0.653)
CRP (AUC 0.71) (95% Cl 0.679 – 0.741)	Lactate (AUC 0.602) (95% Cl 0.554 – 0.649)
LDH (AUC 0.688) (95% Cl 0.654 – 0.721)	BMI (AUC 0.586) (95% Cl 0.542 – 0.63)
Lactate (AUC 0.684) (95% Cl 0.648 – 0.72)	Mg (AUC 0.576) (95% Cl 0.529 – 0.623)
Hb (AUC 0.662) (95% Cl 0.627 – 0.696)	CRP (AUC 0.573) (95% Cl 0.529 – 0.617)
Ferritin (AUC 0.613) (95% Cl 0.578 – 0.648)	LDH (AUC 0.566) (95% Cl 0.517 – 0.615)
BMI (AUC 0.612) (95% Cl 0.581 – 0.651)	SBP (0.53) (95% Cl 0.482 – 0.578)
CPK (AUC 0.576) (95% Cl 0.541 – 0.61)	Ferritin (AUC 0.527) (95% Cl 0.482 – 0.572)
SBP (AUC 0.554) (95% Cl 0.517 – 0.591)	CPK (AUC 0.509) (95% Cl 0.462 – 0.555)

Ordered by highest to lowest AUC.
