# Supplementary material for: Real-time prognostic biomarkers for predicting in-hospital mortality and cardiac complications in COVID-19 patients
Source: PLOS Glob Public Health. 2024 Mar 6;4(3):e0002836. doi: 10.1371/journal.pgph.0002836 (PMC10917247; doi:10.1371/journal.pgph.0002836)
Supplement: S5 Table — (PDF) [file pgph.0002836.s006.pdf]

Table S5. Performance for Biomarkers-Only Model and Full Model for New-Onset Atrial Arrhythmia

Model	AUC (95% CI)	Brier Score	Nagelkerke's R^2	Sensitivity	Specificity
Biomarkers Only	0.728 (0.690, 0.767)	0.071	0.12	0.03	0.997
Full	0.775 (0.741, 0.809)	0.068	0.18	0.036	0.998
