# Supplementary material for: Real-time prognostic biomarkers for predicting in-hospital mortality and cardiac complications in COVID-19 patients
Source: PLOS Glob Public Health. 2024 Mar 6;4(3):e0002836. doi: 10.1371/journal.pgph.0002836 (PMC10917247; doi:10.1371/journal.pgph.0002836)
Supplement: S6 Table — (PDF) [file pgph.0002836.s007.pdf]

Table S6. Coefficients of Full Model for New-Onset Atrial Arrhythmia

Variable	Beta coefficient	95% CI	
Age	0.0433	0.0282	0.0583
Female (Ref: Male)	-0.0303	-0.2024	0.1419
Black race (Ref: White)	0.1246	-0.3164	0.5655
Other race (Ref: White)	-0.6937	-1.4501	0.0627
Diabetes Mellitus	-0.1338	-0.3218	0.0542
Congestive heart failure	-0.0896	-0.3039	0.1248
History of Pulmonary Embolism	-0.2294	-0.7627	0.3038
History of Malignancies[1]	0.00182	-0.2110	0.2146
BMI	0.0102	-0.0134	0.0339
Peak Lactate dehydrogenase (U/L)	0.000026	-0.00031	0.000359
Peak Ferritin (ng/mL)	3.45E-6	-0.00005	0.000059
Peak Troponin-I (ng/mL)	-0.0195	-0.1839	0.1450
Peak Creatine phosphokinase (U/L)	-0.00018	-0.00050	0.000143
Peak C-reactive protein (mg/dL)	-0.00269	-0.0286	0.0232
Peak B-type natriuretic peptide (pg/ml)	0.000463	0.000095	0.000831
Peak Serum Creatinine (mg/dL)	0.0223	-0.0824	0.1271
Peak Lactate (mmol/L)	0.1225	0.0235	0.2215
Peak Serum potassium (mEq/L)	0.4396	0.2209	0.6583
Peak Serum magnesium (mg/dL)	0.0934	-0.3615	0.5483
Lowest Albumin (g/dL)	-0.4142	-0.7508	-0.0776
Lowest Hemoglobin (g/dL)	-0.0299	-0.1295	0.0697
Presenting Systolic blood pressure (mmHg)	-0.00186	-0.00822	0.00450
