# Supplementary material for: Real-time prognostic biomarkers for predicting in-hospital mortality and cardiac complications in COVID-19 patients
Source: PLOS Glob Public Health. 2024 Mar 6;4(3):e0002836. doi: 10.1371/journal.pgph.0002836 (PMC10917247; doi:10.1371/journal.pgph.0002836)
Supplement: S7 Table — (PDF) [file pgph.0002836.s008.pdf]

Table S7. **Coefficients of Biomarker-Only Model for New-Onset Atrial Arrhythmia**

Variable	Beta coefficient	95% CI	
BMI	0.000677	-0.0115	0.0129
Peak Lactate dehydrogenase (U/L)	-0.00018	-0.00049	0.000130
Peak Ferritin (ng/mL)	0.000039	4.577E-6	0.000072
Peak Troponin-I (ng/mL)	0.0183	-0.0671	0.1036
Peak Creatine phosphokinase (U/L)	-0.00003	-0.00011	0.000052
Peak C-reactive protein (mg/dL)	0.00996	-0.00394	0.0239
Peak B-type natriuretic peptide (pg/ml)	0.000515	0.000342	0.000688
Peak Serum Creatinine (mg/dL)	0.0480	0.00352	0.0924
Peak Lactate (mmol/L)	0.0307	-0.0169	0.0783
Peak Serum potassium (mEq/L)	0.2341	0.0983	0.3700
Peak Serum magnesium (mg/dL)	0.1822	-0.0481	0.4126
Lowest Albumin (g/dL)	-0.5955	-0.7908	-0.4002
Lowest Hemoglobin (g/dL)	0.00268	-0.0563	0.0617
Presenting Systolic blood pressure (mmHg)	-0.00275	-0.00655	0.00105
