# Supplementary material for: Real-time prognostic biomarkers for predicting in-hospital mortality and cardiac complications in COVID-19 patients
Source: PLOS Glob Public Health. 2024 Mar 6;4(3):e0002836. doi: 10.1371/journal.pgph.0002836 (PMC10917247; doi:10.1371/journal.pgph.0002836)
Supplement: S8 Table — (PDF) [file pgph.0002836.s009.pdf]

Table S8. **Odds Ratios of Biomarker-Only Model for ICU Admission**

Variable	OR	95% CI	
BMI	1.026	1.016	1.036
Peak Lactate dehydrogenase (U/L)	1.001	1.001	1.002
Peak Ferritin (ng/mL)	1.000	1.000	1.000
Peak Troponin-I (ng/mL)	1.064	0.996	1.137
Peak Creatine phosphokinase (U/L)	1.000	1.000	1.000
Peak C-reactive protein (mg/dL)	0.999	0.986	1.011
Peak B-type natriuretic peptide (pg/ml)	1.000	1.000	1.000
Peak Serum Creatinine (mg/dL)	0.998	0.948	1.030
Peak Lactate (mmol/L)	1.221	1.153	1.293
Peak Serum potassium (mEq/L)	1.790	1.582	2.026
Peak Serum magnesium (mg/dL)	4.480	3.515	5.709
Lowest Albumin (g/dL)	0.327	0.276	0.387
Lowest Hemoglobin (g/dL)	0.887	0.844	0.932
Presenting Systolic blood pressure (mmHg)	1.001	0.997	1.004
