# Supplementary material for: Real-time prognostic biomarkers for predicting in-hospital mortality and cardiac complications in COVID-19 patients
Source: PLOS Glob Public Health. 2024 Mar 6;4(3):e0002836. doi: 10.1371/journal.pgph.0002836 (PMC10917247; doi:10.1371/journal.pgph.0002836)
Supplement: S9 Table — (PDF) [file pgph.0002836.s010.pdf]

Table S9. Odds Ratios of Biomarker-Only Model for Renal Failure

Variable	OR	95% CI	
BMI	1.026	1.012	1.039
Peak Lactate dehydrogenase (U/L)	1.000	1.000	1.000
Peak Ferritin (ng/mL)	1.000	1.000	1.000
Peak Troponin-I (ng/mL)	1.039	0.947	1.140
Peak Creatine phosphokinase (U/L)	1.000	1.000	1.000
Peak C-reactive protein (mg/dL)	1.036	1.018	1.054
Peak B-type natriuretic peptide (pg/ml)	1.000	1.000	1.000
Peak Lactate (mmol/L)	1.079	0.995	1.169
Peak Serum potassium (mEq/L)	1.684	1.425	1.990
Peak Serum magnesium (mg/dL)	2.402	1.775	3.250
Lowest Albumin (g/dL)	0.634	0.509	0.789
Lowest Hemoglobin (g/dL)	0.976	0.914	1.042
Presenting Systolic blood pressure (mmHg)	0.991	0.986	0.995
