# Supplementary material for: Real-time prognostic biomarkers for predicting in-hospital mortality and cardiac complications in COVID-19 patients
Source: PLOS Glob Public Health. 2024 Mar 6;4(3):e0002836. doi: 10.1371/journal.pgph.0002836 (PMC10917247; doi:10.1371/journal.pgph.0002836)
Supplement: S1 Fig — (PDF) [file pgph.0002836.s011.pdf]

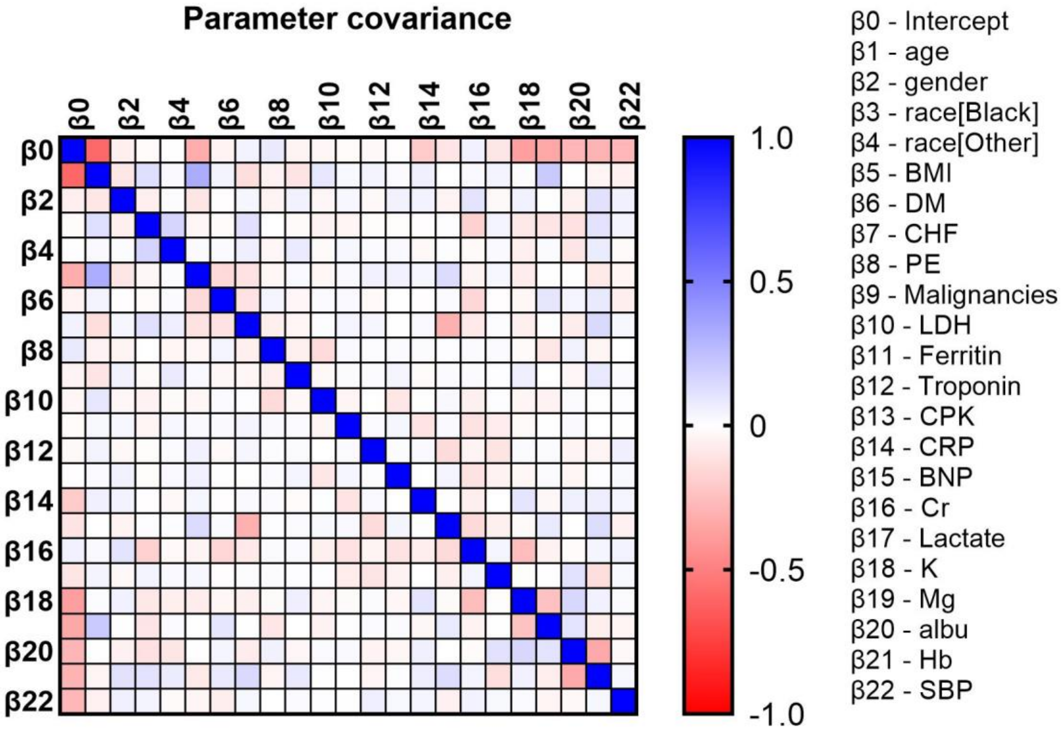

Fig S1. Parameter Covariance Heatmap

1 (blue) represents perfect positive correlation and -1 (red) represents perfect negative correlation
