# Supplementary material for: Real-time prognostic biomarkers for predicting in-hospital mortality and cardiac complications in COVID-19 patients
Source: PLOS Glob Public Health. 2024 Mar 6;4(3):e0002836. doi: 10.1371/journal.pgph.0002836 (PMC10917247; doi:10.1371/journal.pgph.0002836)
Supplement: S2 Fig — (PDF) [file pgph.0002836.s012.pdf]

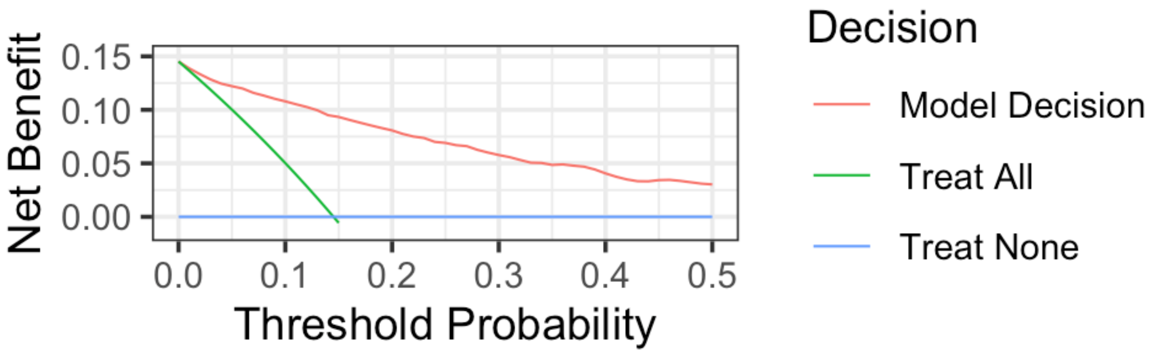

Fig S2. Decision Curve Analysis for Full Model for In-Hospital Mortality

Net benefit is calculated as $\frac{true\ positives}{N} - \frac{false\ positives}{N} * \frac{p_t}{1-p_t}$ where p_t is the threshold probability to define when a patient is positive and N is the total sample size. Positive is defined as predicted death.
