# Supplementary material for: Real-time prognostic biomarkers for predicting in-hospital mortality and cardiac complications in COVID-19 patients
Source: PLOS Glob Public Health. 2024 Mar 6;4(3):e0002836. doi: 10.1371/journal.pgph.0002836 (PMC10917247; doi:10.1371/journal.pgph.0002836)
Supplement: S3 Fig — (PDF) [file pgph.0002836.s013.pdf]

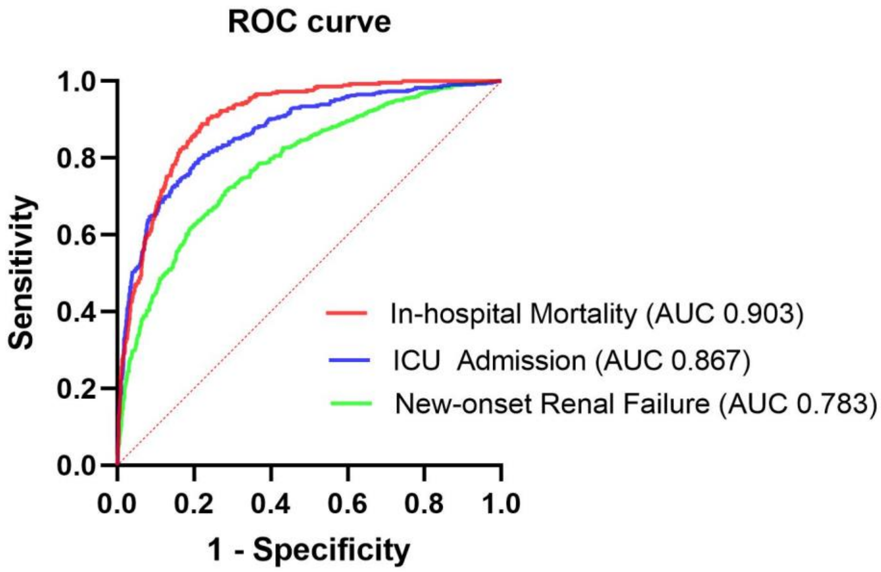

Fig S3. ROC for Model Adjusting for Type of Atrial Arrhythmia

Type of atrial arrhythmia (AA) included past history of AA and new onset of AA
