# Supplementary material for: Real-time prognostic biomarkers for predicting in-hospital mortality and cardiac complications in COVID-19 patients
Source: PLOS Glob Public Health. 2024 Mar 6;4(3):e0002836. doi: 10.1371/journal.pgph.0002836 (PMC10917247; doi:10.1371/journal.pgph.0002836)
Supplement: S4 Fig — (PDF) [file pgph.0002836.s014.pdf]

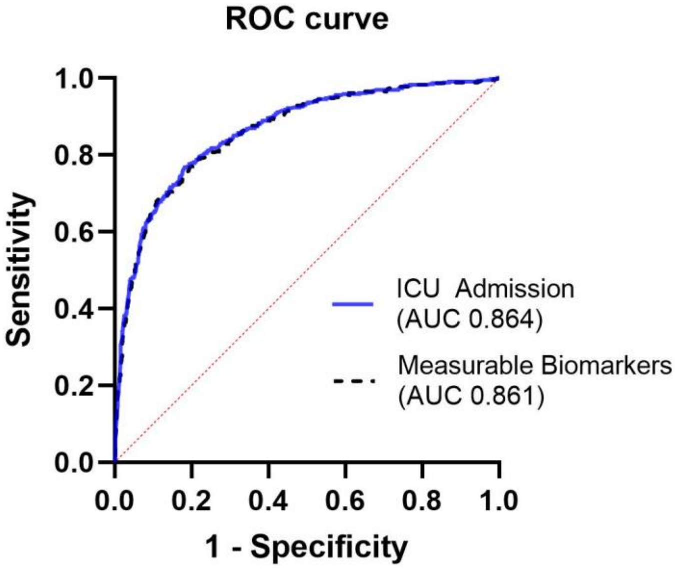

Fig S4. ROC for ICU Admission*

* Fig S4 and Fig S5: Demographics (age, gender, race), Medical history (Diabetes mellitus, Congestive heart failure, Pulmonary Embolism, Malignancies), Measurable biomarkers (BMI, LDH, Ferritin, Troponin, Creatinine phosphokinase (CPK), C-reactive protein (CRP), B-type Natriuretic Peptide (BNP), Creatinine (Cr), Lactate, Potassium (K), Magnesium (Mg), Albumin, Hemoglobin (Hb), Systolic Blood Pressure (SBP) were used to predict outcome ICU admission (Fig S4) and all above excluding Cr were used to predict outcome New-onset Renal Failure (Fig S5).
